# Soil chemical attributes in areas under conversion from forest to pasture in southern Brazilian Amazon

**DOI:** 10.1038/s41598-022-25406-9

**Published:** 2022-12-29

**Authors:** Alan Ferreira Leite de Lima, Milton César Costa Campos, Thalita Silva Martins, Guilherme Abadia Silva, Wildson Benedito Mendes Brito, Luís Antônio Coutrim dos Santos, Ivanildo Amorim de Oliveira, José Maurício da Cunha

**Affiliations:** 1 Amazon Environment and Soil Research Group (GPSAA), Faculty of Agrarian Sciences, Federal University of Amazonas (FCA/UFAM), Manaus, AM Brazil; 2 Amazon Environment and Soil Research Group (GPSAA), Department of Soils and Rural Engineering, Agricultural Sciences Center, Federal University of Paraíba (DSER/CCA/UFPB), Areia, PB Brazil; 3 Amazon Environment and Soil Research Group (GPSAA), Institute of Education, Agriculture and Environment, Federal University of Amazonas (IEAA/UFAM), Humaitá, AM Brazil; 4 Amazon Environment and Soil Research Group (GPSAA), Higher Study Center of Itacoatiara, Amazonas State University (CESIT/UEA), Itacoatiara, AM Brazil; 5 Amazon Environment and Soil Research Group (GPSAA), Federal Institute of Education, Science and Technology of Rondônia (IFRO), Ariquemes, RO Brazil

**Keywords:** Environmental sciences, Environmental impact

## Abstract

The south of the Brazilian Amazon is one of the largest cattle-producing regions in Brazil, however, most of the pastures are in low fertility soils. Thus, cattle breeders compensate for the low production of pastures, increasing the size of the areas, generating more deforestation and burning. These practices increase the chemical degradation process of Amazonian soils, making them increasingly infertile when improperly managed. With this, the objective of the work was to evaluate the impacts caused in the chemical attributes of soils, in areas under forest-to-pasture conversion, in the south of the Brazilian Amazon. The study was carried out in the district of União Bandeirantes, in an area of forest and two areas with pastures (brachiaria and mombaça grass). In the field, soil samples were collected at two depths (0.00–0.10 and 0.10–0.20 m), to carry out chemical analyzes. Further, uni, bi and multivariate statistical analyzes were carried out, besides geostatistical analyzes were carried out to study spatial variability and management zones. The conversion of forest to pasture increased the pH and exchangeable bases levels, reducing the availability of exchangeable aluminum and potential acidity, however, it induces losses of phosphorus and organic carbon from the soil. Among the pasture environments, the mombaça grass area presented higher fertility. Greater spatial variability of chemical attributes was observed in the environment with mombaça grass, indicating greater heterogeneity in the distribution of attributes in the area. We attribute this behavior to the higher grazing intensity and the micro-reliefs in the area that direct the flow of water and nutrients.

## Introduction

The state of Rondônia is closely linked to agricultural and livestock production, among which the cattle production stands out, being the fifth largest producer among the Brazilian states with 13,871,863 bovine heads. Porto Velho, the state capital, is an important cattle producer, occupying the seventh place among the municipalities with 968,778 heads^1^. However, studies show that pasture areas are becoming degraded over time, due to the low management adopted^2^, causing losses in the production of milk and meat. Therefore, studies in the region that assess soil fertility under pastures are necessary to provide information on the impacts generated and which attributes are most sensitive.

Among commercial crops, forage species represent the most cultivated plants of economic interest in Brazil and in the world^3^. Among the forage plants used by animals, grasses of the genus Brachiaria and Panicum are the main options for feeding the Brazilian cattle herd^4^.

Slash-and-burn practice has been a cheap alternative to clear forest areas and introduce pastures in the Amazon^5^. After its use, if the soil is not managed correctly, it can cause negative changes in its attributes, and consequently, degradation. In this case, the more inclined relief can intensify them^6^. The relief, or topographic position, is capable of altering the water content of the soil and the intensity of the processes of removal and deposition of sediments (erosion)^7^, increasing the risks of erosion and loss of nutrients^8^. In studies evaluating forest-pasture conversion, Araújo et al.^9^ and Lisbôa et al.^10^ showed that the most sensitive chemical attributes are organic matter, Ca^2+^, Mg^2+^, K^+^ and P.

In pasture systems, when properly managed, they can improve many soil properties, such as water retention, soil organic matter and nutrient cycling^11^. Braz et al.^12^ observed an increase in soil carbon stocks after 8 years of use with pastures in relation to the forest, and that, if fire is used frequently for cleaning, there is a decrease. In addition, they found that the conversion of forest to pasture with the use of fire increases the pH values and availability of P, Ca^2+^ and K^+^ in the soil, and decreases the exchangeable Al^3+^.

Although there are several studies that have evaluated the impacts caused on the soil after converting forest environments to pastures^2,5,12,13^ studies are still needed in the Rondônia region, which seek to increase forage production, only increasing soil fertility, eliminating the demand for deforestation. With this, the objective was to evaluate the impacts caused on the chemical attributes of soils, in areas of forest conversion in pastures, in the south of the Brazilian Amazon.

## Results

### Conversion of forest to pasture

When evaluating the dispersion of the variables (Table 1), we observed that the coefficient of variation (CV), presented values varying from 6.71 to 65.37%, being classified as low (< 12%), medium (12–24%) and high (> 60%)^14^. The pH in all areas and V% at the depth of 0.10–0.20 m in the forest area showed CV classified as low. Ca^2+^ at a depth of 0.00–0.10 m, Na^+^ at a depth of 0.10–0.20 m in the area of brachiaria and mombaça grass, and K^+^ at a depth of 0.10–0.20 m in the area of mombaça presented CV classified as high. The other variables presented CV classified as moderate for all areas and depths evaluated.

**Table 1:** Descriptive analysis and data normality test for chemical attributes in environments under conversion, in Porto Velho, Rondônia.

Parameters	pH	Al^3+^	H + Al	Ca^2+^	Mg^2+^	K^+^	Na^+^	CEC	V	P	TOC
	H_2_O	cmol_c_ kg^−1^							%	mg kg^−1^	g kg^−1^
**Forest**											
Depth 0.00–0.10 m											
CV	9.06	17.72	12.67	26.19	36.60	23.33	43.33	12.15	14.43	23.92	17.49
Skewness	0.97	0.33	0.61	0.43	0.88	0.06	0.53	0.44	− 0.27	0.59	0.36
Kurtosis	1.40	1.06	0.27	− 0.96	0.44	− 0.74	− 0.59	0.55	− 0.86	0.15	− 0.09
KS	0.09*	0.12*	0.13*	0.13*	0.11*	0.09*	0.18^ ns^	0.10*	0.12*	0.07*	0.08*
Depth 0.10–0.20 m											
CV	6.71	14.03	13.17	12.02	37.23	31.56	42.37	13.74	9.49	25.90	14.05
Skewness	0.96	0.87	0.18	0.97	0.56	0.82	− 0.03	0.37	0.19	0.11	0.43
Kurtosis	0.32	2.43	− 0.43	0.52	− 0.42	0.87	− 1.49	− 0.37	− 0.16	− 1.22	− 0.31
KS	0.16*	0.10*	0.11*	0.16*	0.11*	0.13*	0.22^ ns^	0.11*	0.10*	0.12*	0.09*
**Brachiaria**											
Depth 0.00–0.10 m											
CV	10.02	46.33	30.23	65.37	43.44	30.15	53.76	48.48	48.54	36.70	22.64
Skewness	0.40	0.57	0.05	0.76	0.40	0.45	0.70	0.44	0.57	0.53	0.77
Kurtosis	1.05	− 0.70	− 1.12	− 0.44	− 0.60	− 0.58	− 0.10	− 0.17	− 0.58	− 0.10	0.56
KS	0.07*	0.17^ ns^	0.11*	0.13*	0.10*	0.11*	0.14*	0.09*	0.10*	0.11*	0.07*
Depth 0.10–0.20 m											
CV	8.63	37.44	31.05	53.14	57.95	48.89	69.47	24.34	55.62	48.10	23.64
Skewness	− 0.20	0.57	0.44	0.59	0.79	1.13	0.31	0.56	0.62	0.62	− 0.46
Kurtosis	1.63	0.34	− 0.67	− 1.11	− 0.73	0.70	− 0.71	− 0.53	− 0.81	− 0.51	− 0.23
KS	0.11*	0.11*	0.13*	0.20^ ns^	0.16^ ns^	0.17^ ns^	0.18^ ns^	0.12*	0.14*	0.12*	0.09*
**Mombaça**											
Depth 0.00–0.10 m											
CV	8.84	35.72	22.52	42.92	36.54	50.64	54.51	32.31	42.95	38.94	22.60
Skewness	0.29	− 0.51	0.41	0.66	1.02	0.32	0.63	0.45	0.20	0.56	0.17
Kurtosis	− 0.46	− 0.93	0.48	− 0.43	0.30	− 1.16	− 0.67	− 0.70	− 0.94	− 0.87	− 0.32
KS	0.07*	0.20^ ns^	0.10*	0.14*	0.13*	0.16*	0.22^ ns^	0.10*	0.10*	0.12*	0.09*
Depth 0.10–0.20 m											
CV	7.51	25.51	23.73	44.49	21.17	64.55	85.28	17.69	29.91	40.56	23.55
Skewness	1.03	− 0.18	0.08	1.33	1.13	1.25	0.53	0.38	1.03	1.10	0.39
Kurtosis	0.77	− 0.35	0.09	1.41	1.63	1.06	− 1.19	− 0.47	0.58	0.80	− 0.17
KS	0.14*	0.10*	0.09*	0.14*	0.13*	0.20^ ns^	0.31^ ns^	0.10*	0.16*	0.16*	0.07*

*CV* Coefficient of variation, *KS* Kolmogorov–Smirnov; ^ns^ = not significant at 5% probability; * significant at 5% probability.

**Figure 1:**
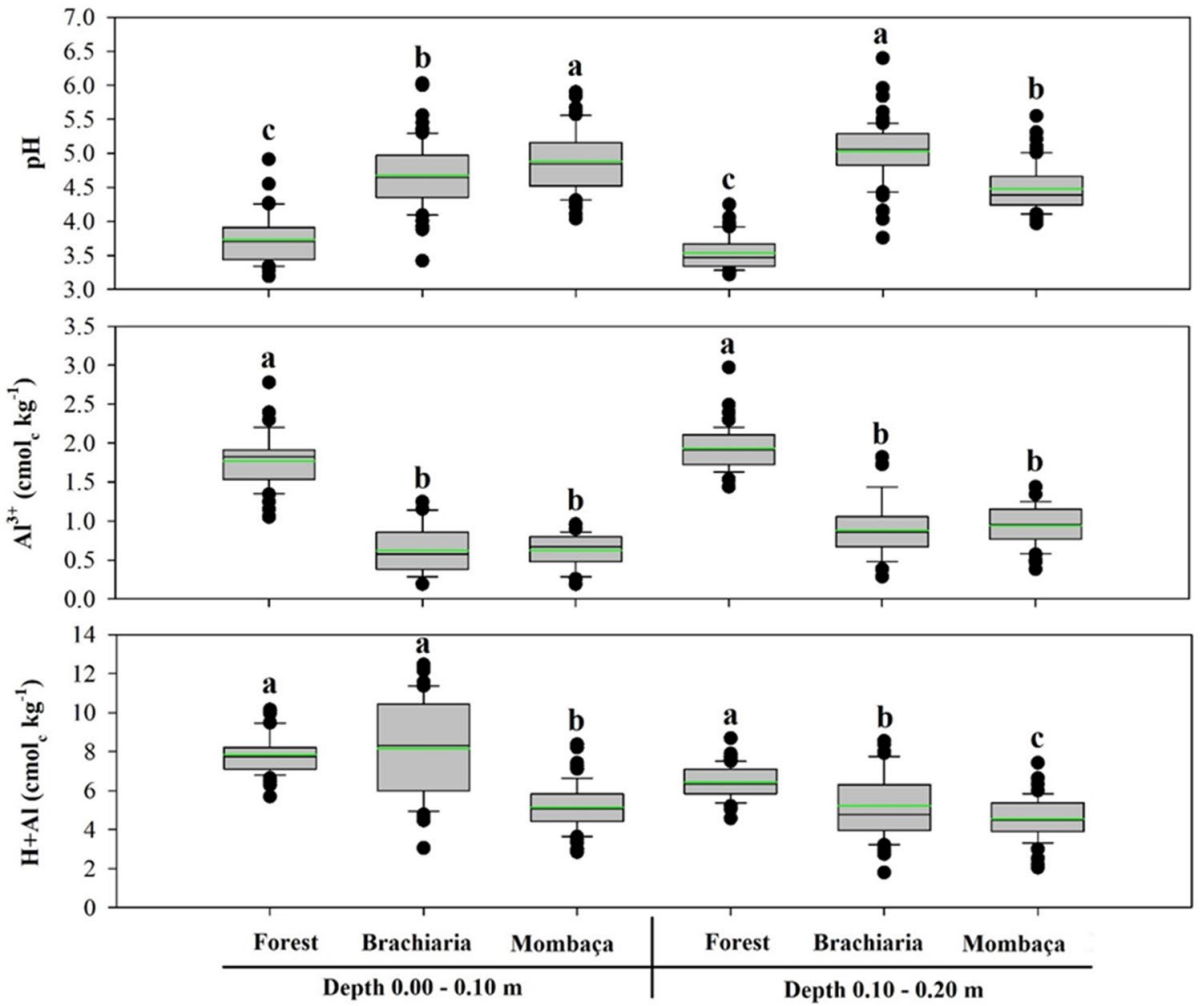
Boxplot and mean test of soil acidity components in different environments, in Porto Velho, Rondônia. The green line indicates the mean value of the data. Different lower case letters represent that there were significant differences by the Tukey test at 5%.

In the components of soil acidity, it can be seen that there was a statistical difference for all variables (Fig. 1). For the pH, the area with mombaça showed the highest values at the depth 0.00–0.10 m (up to 4.88); however, at the depth of 0.10–0.20 m it was observed in the area with brachiaria values up to 5.03. The lowest values were found in the forest area at both depths (3.73 and 3.53). The highest levels of Al^3+^ were obtained in the forest area at both depths (1.77 and 1.93 cmol_c_ kg^−1^), while the pasture areas showed the lowest levels, with values between 0.62 and 0.94 cmol_c_ kg^−1^, not differing statistically from each other.

The highest H + Al content was observed in 0.00–0.10 m of brachiaria (8.16 cmol_c_ kg^−1^) and in the forest area at a depth of 0.10–0.20 m (6.44 cmol_c_ kg^−1^), however, the mombaça grass area had the lowest levels (5.15 and 4.55 cmol_c_ kg^−1^) in the two depths studied.

**Figure 2:**
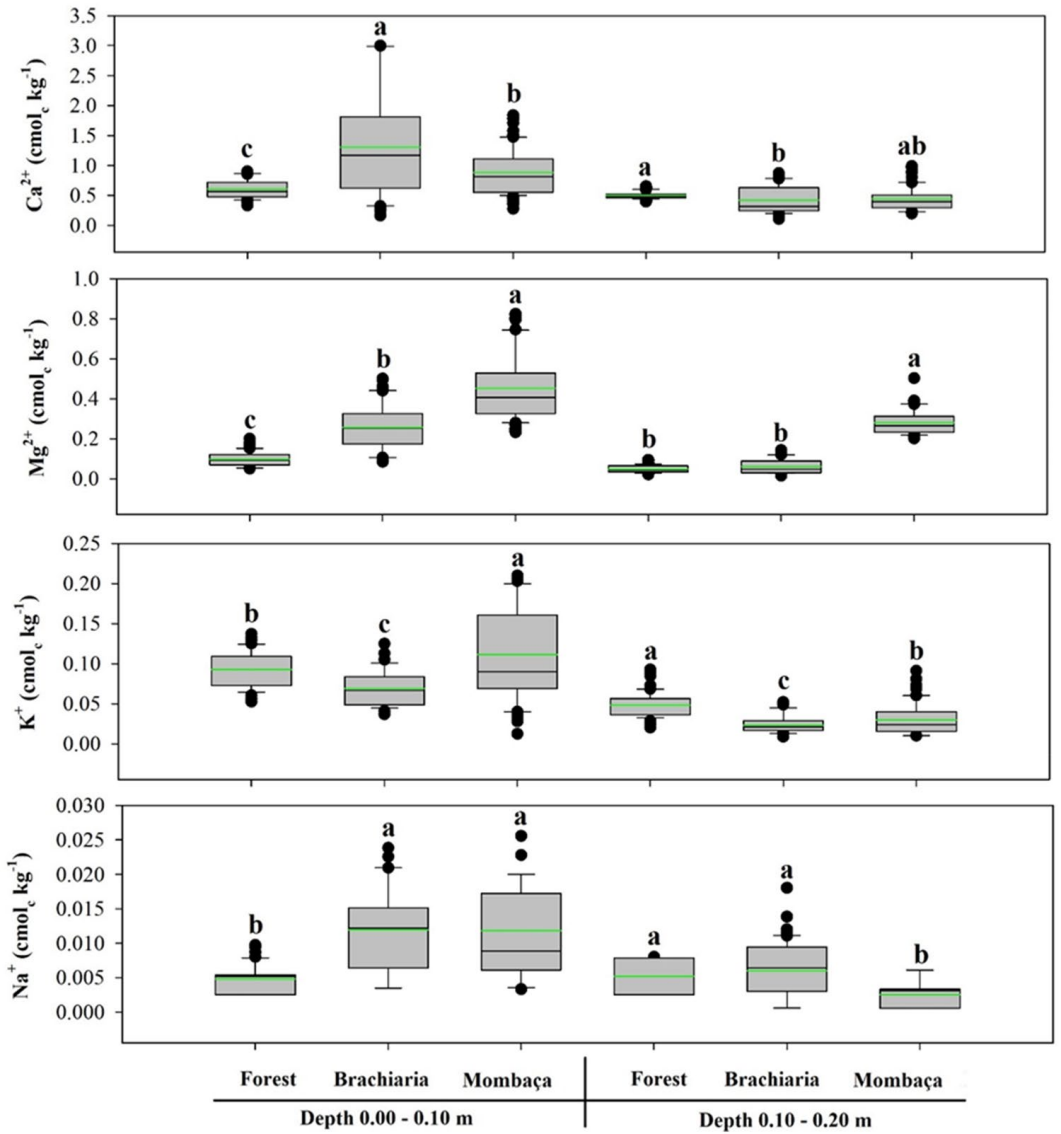
Boxplot and mean test of exchangeable soil bases in different environments, in Porto Velho, Rondônia. The green line indicates the mean value of the data. Different lower case letters represent that there were significant differences by the Tukey test at 5%.

The exchangeable bases showed a significant difference for the areas at the different depths studied (Fig. 2). The highest levels of bases were found for pasture areas in relation to the forest at a depth of 0.00–0.10 m, a brachiaria area with the highest levels of Ca^2+^ and Na^+^ (1.30 and 0.0119 cmol_c_ kg^−1^), while the area with mombaça grass has the highest levels of Mg^2+^ and K^+^ (0.45 and 0.11 cmol_c_ kg^−1^). At a depth of 0.10–0.20 m, the forest area showed the highest levels of Ca^2+^, K^+^ and Na^+^ (0.5, 0.05 and 0.005 cmol_c_ kg^−1^, respectively), while the mombaça grass area presented the highest levels of Mg^2+^ (0.28 cmol_c_ kg^−1^).

**Figure 3:**
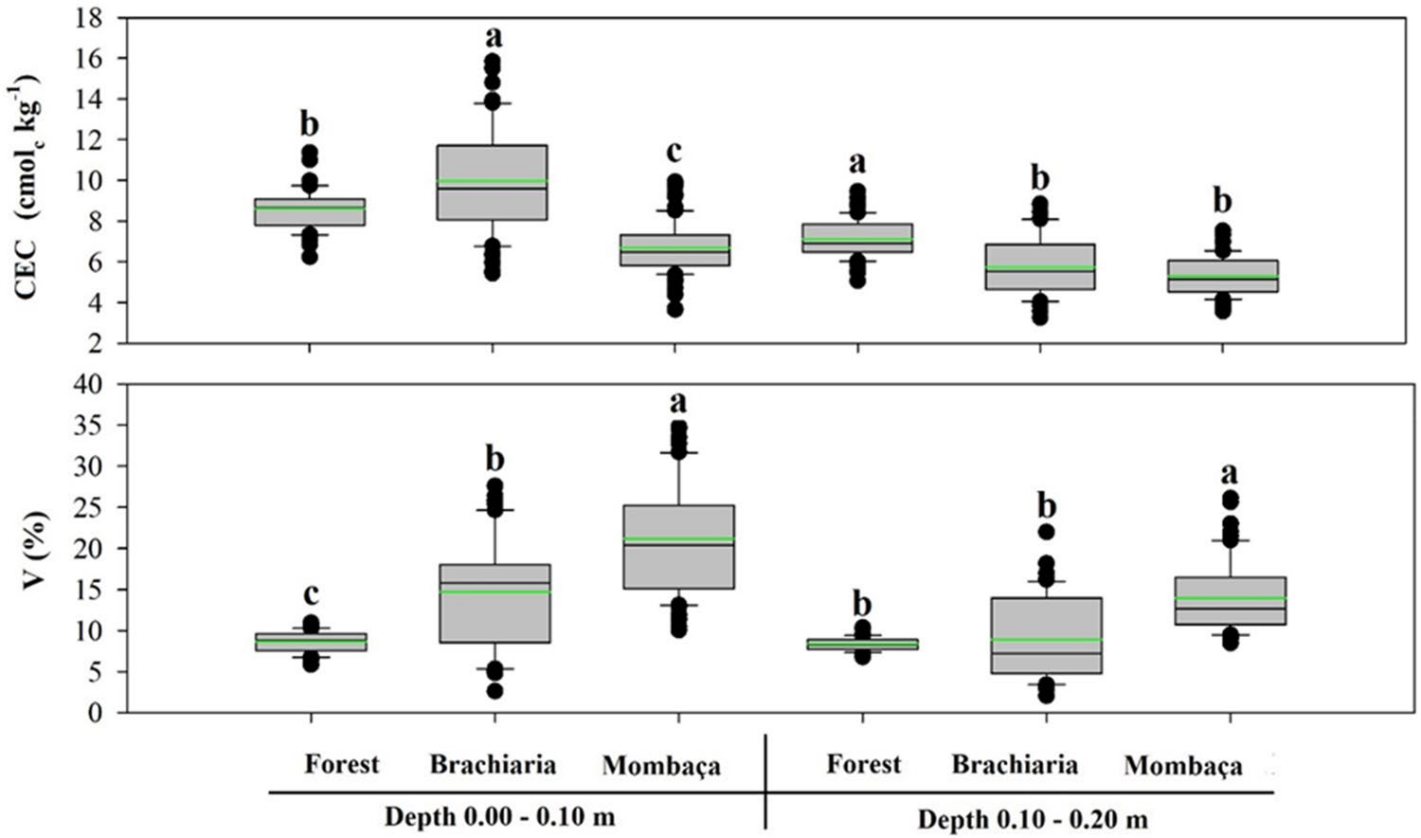
Boxplot and mean test of cation exchange capacity and saturation by soil bases in different environments, in Porto Velho, Rondônia. The green line indicates the mean value of the data. Different lowercase letters represent that there were significant differences by the Tukey test at 5%.

At the 0.00–0.10 m depth, the CEC showed a significant difference, in which the highest values were seen in the brachiaria area (9.80 cmol_c_ kg^−1^) and the lowest for mombaça grass area (6.61 cmol_c_ kg^−1^). At a depth of 0.10–0.20 m, the highest values were observed for the forest area (7.04 cmol_c_ kg^−1^) and the lowest values for mombaça grass area (5.29 cmol_c_ kg^−1^) (Fig. 3). The mombaça grass area showed the highest values of base saturation at the two depths studied (22.07 and 14.12 cmol_c_ kg^−1^) (Fig. 3).

**Figure 4:**
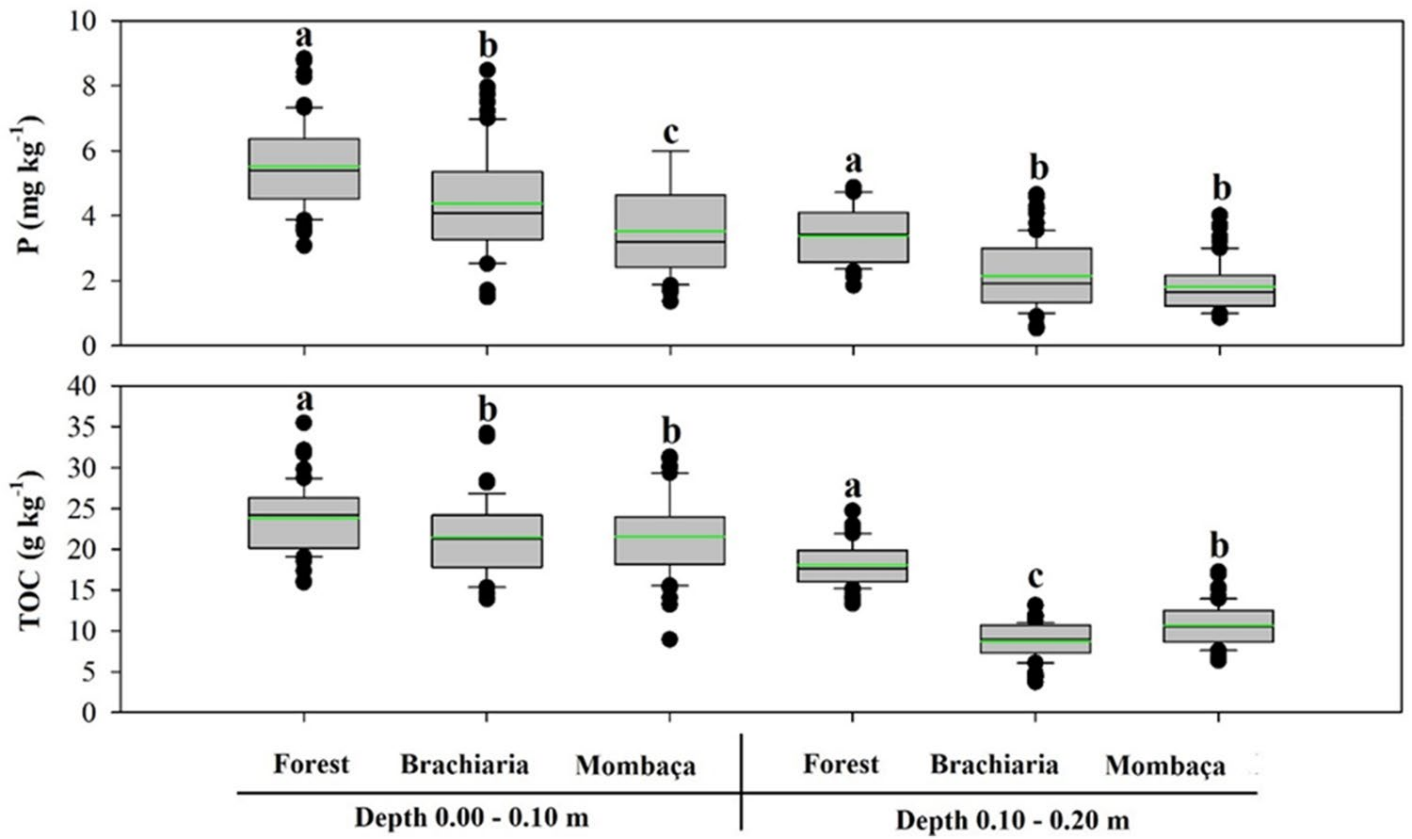
Boxplot and mean test for phosphorus and total organic carbon of the soil in different environments, in Porto Velho, Rondônia. The green line indicates the mean value of the data. Different lower case letters represent that there were significant differences by the Tukey test at 5%.

The forest area showed the highest levels of P (5.52 and 3.39 mg kg^−1^) and TOC (23.88 and 18.09 g kg^−1^) in the two depths studied in relation to pasture environments (Fig. 4). Among the pasture areas, mombaça grass had the lowest levels of P (3.53 and 1.81 mg kg^−1^) and the highest levels of TOC (21.58 and 10.68 g kg^−1^).

### Interactions between environments and soil chemical attributes

The interactions between each attribute for the environments studied are shown in Table 2. It is observed that the pH showed a significant positive correlation with Ca^2+^, Mg^2+^, V%, and negative for Al^3+^ in all areas, and also negative for H + Al in pasture environments. Al^3+^ showed a negative correlation with exchangeable bases for all areas studied. In addition, we observed a significant positive correlation of pH with K^+^ in the forest and mombaça area, and with Na^+^ and CEC for mombaça grass area. The brachiaria area, on the other hand, showed a negative correlation between pH and CEC. CEC also showed a positive and high correlation with H + Al (0.86–0.99) for all areas studied.

**Table 2:** Pearson’s correlations of soil chemical attributes in different environments, in Porto Velho, Rondônia.

	pH	Al^3+^	H + Al	Ca^2+^	Mg^2+^	K^+^	Na^+^	CEC	V%	P	TOC
**Forest**											
pH	1.00	− 0.47**	0.10^ ns^	0.34**	0.29**	0.31**	− 0.16^ ns^	0.15^ ns^	0.36**	0.32**	0.26**
Al^3+^	− 0.47**	1.00	0.14^ ns^	− 0.25**	− 0.25**	− 0.17^ ns^	0.12^ ns^	0.08^ ns^	− 0.37**	− 0.09^ ns^	− 0.06^ ns^
H + Al	0.10^ ns^	0.14^ ns^	1.00	0.42**	0.26**	0.66**	− 0.12^ ns^	0.99**	− 0.06 ns	0.61**	0.84**
Ca^2+^	0.34**	− 0.25**	0.42**	1.00	0.62**	0.67**	− 0.05^ ns^	0.55**	0.83**	0.41**	0.55**
Mg^2+^	0.29**	− 0.25**	0.26**	0.62**	1.00	0.55**	− 0.05^ ns^	0.37**	0.76**	0.32**	0.41**
K^+^	0.31**	− 0.17^ ns^	0.66**	0.67**	0.55**	1.00	− 0.01^ ns^	0.73**	0.46**	0.72**	0.73**
Na^+^	− 0.16^ ns^	0.12^ ns^	− 0.12^ ns^	− 0.05^ ns^	− 0.05^ ns^	− 0.01^ ns^	1.00	− 0.12^ ns^	0.01^ ns^	− 0.07^ ns^	− 0.05^ ns^
CEC	0.15^ ns^	0.08^ ns^	0.99**	0.55**	0.37**	0.73**	− 0.12^ ns^	1.00	0.10^ ns^	0.64**	0.87**
V%	0.36**	− 0.37**	− 0.06^ ns^	0.83**	0.76**	0.46**	0.01^ ns^	0.10^ ns^	1.00	0.18^ ns^	0.20*
P	0.32**	− 0.09^ ns^	0.61**	0.41**	0.32**	0.72**	− 0.07^ ns^	0.64**	0.18^ ns^	1.00	0.64**
TOC	0.26**	− 0.06^ ns^	0.84**	0.55**	0.41**	0.73**	− 0.05^ ns^	0.87**	0.20*	0.64**	1.00
**Brachiaria**											
pH	1.00	− 0.41**	− 0.50**	0.32**	0.24**	− 0.01^ ns^	− 0.01^ ns^	− 0.36**	0.51**	0.03^ ns^	− 0.27**
Al^3+^	− 0.41**	1.00	0.02^ ns^	− 0.56**	− 0.48**	− 0.37**	− 0.12^ ns^	− 0.17*	− 0.57**	− 0.23**	− 0.32**
H + Al	− 0.50**	0.02^ ns^	1.00	0.12^ ns^	0.19*	0.23**	0.03^ ns^	0.95**	− 0.31**	0.22*	0.57**
Ca^2+^	0.32**	− 0.56**	0.12^ ns^	1.00	0.81**	0.40**	0.10^ ns^	0.42**	0.84**	0.43**	0.66**
Mg^2+^	0.24**	− 0.48**	0.19*	0.81**	1.00	0.45**	0.05^ ns^	0.44**	0.69**	0.39**	0.60**
K^+^	− 0.01^ ns^	− 0.37**	0.23**	0.40**	0.45**	1.00	0.19*	0.35**	0.34**	0.33**	0.50**
Na^+^	− 0.01^ ns^	− 0.12^ ns^	0.03^ ns^	0.11^ ns^	0.05^ ns^	0.19*	1.00	0.07^ ns^	0.11^ ns^	0.02^ ns^	0.17*
CEC	− 0.36**	− 0.17*	0.95**	0.42**	0.44**	0.35**	0.07^ ns^	1.00	− 0.01n^ns^	0.33**	0.73**
V%	0.51**	− 0.57**	− 0.31**	0.84**	0.69**	0.34**	0.11^ ns^	− 0.01^ ns^	1.00	0.25**	0.37**
P	0.03^ ns^	− 0.23**	0.22*	0.43**	0.39**	0.33**	0.02^ ns^	0.33**	0.25**	1.00	0.46**
TOC	− 0.27**	− 0.32**	0.57**	0.66**	0.60**	0.50**	0.17*	0.73**	0.37**	0.46**	1.00
**Mombaça**											
pH	1.00	− 0.76**	− 0.18*	0.76**	0.71**	0.47**	0.34**	0.26**	0.85**	0.51**	0.49**
Al^3+^	− 0.76**	1.00	0.06^ ns^	− 0.70**	− 0.66**	− 0.49**	− 0.38**	− 0.33**	− 0.75**	− 0.43**	− 0.46**
H + Al	− 0.18*	0.06^ ns^	1.00	0.01^ ns^	0.11^ ns^	0.13^ ns^	0.10^ ns^	0.86**	− 0.39**	0.26**	0.35**
Ca^2+^	0.76**	− 0.70**	0.01^ ns^	1.00	0.73**	0.42**	0.29**	0.52**	0.85**	0.52**	0.48**
Mg^2+^	0.71**	− 0.66**	0.11^ ns^	0.73**	1.00	0.60**	0.34**	0.53**	0.71**	0.58**	0.59**
K^+^	0.47**	− 0.49**	0.13^ ns^	0.42**	0.60**	1.00	0.51**	0.41**	0.47**	0.40**	0.59**
Na^+^	0.34**	− 0.38**	0.10^ ns^	0.29**	0.34**	0.51**	1.00	0.27**	0.31**	0.33**	0.48**
CEC	0.26**	− 0.33**	0.86**	0.52**	0.53**	0.41**	0.27**	1.00	0.13^ ns^	0.51**	0.59**
V%	0.85**	− 0.75**	− 0.39**	0.85**	0.71**	0.47**	0.31**	0.13n^ns^	1.00	0.42**	0.41**
P	0.51**	− 0.43**	0.26**	0.52**	0.58**	0.40**	0.33**	0.51**	0.42**	1.00	0.60**
TOC	0.49**	− 0.46**	0.35**	0.48**	0.59**	0.59**	0.48**	0.59**	0.41**	0.60**	1.00

^ns^ = not significant at 5% probability; * and ** significant at 5 and 1% probability.

The total organic carbon (TOC) of the soil showed a significant positive correlation with Ca^2+^, Mg^2+^, K^+^, V%, CEC and P in all areas studied (Table 2). A positive correlation was observed between TOC and P, indicating that P is directly related to TOC and, consequently, organic matter. The TOC presented a positive correlation with pH in the forest and mombaça areas, however, a negative correlation with pH in the brachiaria area. Al^3+^ showed a negative correlation with TOC in pasture areas.

**Figure 5:**
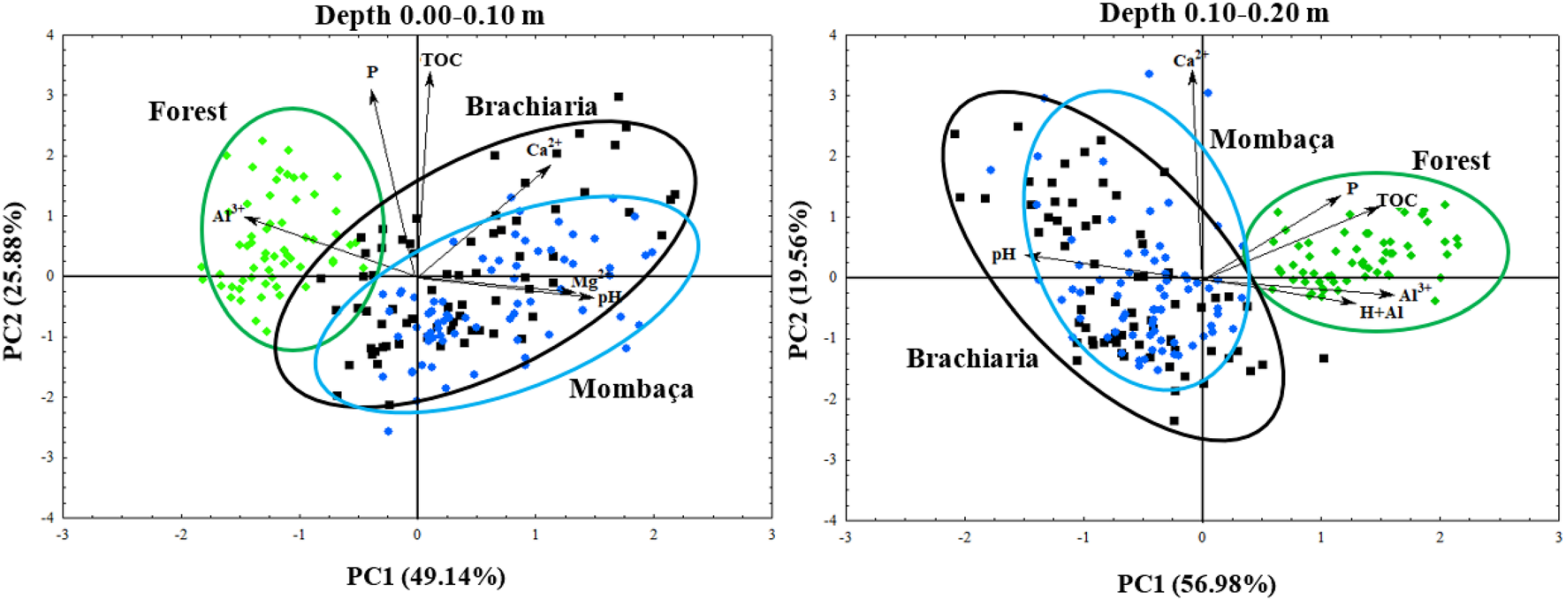
Factorial plane of the chemical attributes of the soil in forest and pasture areas, in Porto Velho, Rondônia.

Factor analysis showed significant results for the depth 0.00–0.10 and 0.10–0.20 m (KMO = 0.714 and 0.777 and *p* < 0.05 for the Barlett sphericity test), for the variables studied in the different environments, showing suitability for the construction of the Principal Components (PC), allowing to transform 11 original variables into two factors (Fig. 5) (Table 3).

**Table 3:** Correlation between each principal component with the chemical attributes of the soil in forest and pastures areas, in Porto Velho, Rondônia.

Attributes	Depth 0.00–0.10 m			Depth 0.10–0.20 m		
	Common variance	Factors		Common variance	Factors	
		PC1	PC2		PC1	PC2
pH	0.78	0.93*	− 0.08	0.75	− 0.88*	0.10
Al^3+^	0.76	− 0.90*	0.24	0.79	0.92*	-0.08
H + Al	–	–	**–**	0.40	0.75*	-0.11
Ca^2+^	0.48	0.70*	0.46	0.30	− 0.05	0.96*
Mg^2+^	0.60	0.84*	− 0.06	–	–	–
P	0.26	− 0.23	0.77*	0.41	0.68*	0.38
TOC	0.32	0.06	0.84*	0.75	0.86*	0.33
Explanatory variance (%)		49.14	25.88	–	56.98	19.56

*More discriminatory values.

*PC1* Principal component 1, *PC2* Principal component 2.

At a depth of 0.00–0.10 m, both factors were responsible for explaining 75.02% of the variance of variables with eigenvalues greater than 1. PC1 explains 49.14%, being responsible for the variables pH, Al^3+^, Ca^2+^ and Mg^2+^, PC2 explains 25.88% of the variance, being responsible for P and TOC. In PC1, pH, Ca^2+^ and Mg^2+^ showed positive values, while Al^3+^ presented negative values, this indicates that the attributes that showed the same signs have a direct correlation while those that have opposite signs have an inverse correlation. At PC2, P and TOC showed positive values (Table 3). When observing the factorial plane for a depth of 0.00–0.10 m, two groups are formed, the first being formed by the forest, which is discriminated by Al^3+^, P and TOC, which have eigenvalues greater than the mean, and the second formed by pastures areas that are characterized by high pH, Ca^2+^ and Mg^2+^ values (Fig. 5).

At a depth of 0.10–0.20 m, both factors were responsible for explaining 76.54% of the explanatory variance. PC1 explained 56.98% being responsible for P, pH, Al^3+^, H + Al and TOC, PC2 explained 19.56% being responsible for Ca^2+^. In PC1, most attributes showed a positive value while the pH showed a negative value (Table 3). Through the factorial plan, two groups are formed, group one being formed by the forest, which is discriminated by Al^3+^, H + Al, P and TOC, while group two is composed of pastures that are discriminated by pH and Ca^2+^ (Fig. 5).

### Spatial variability

**Figure 6:**
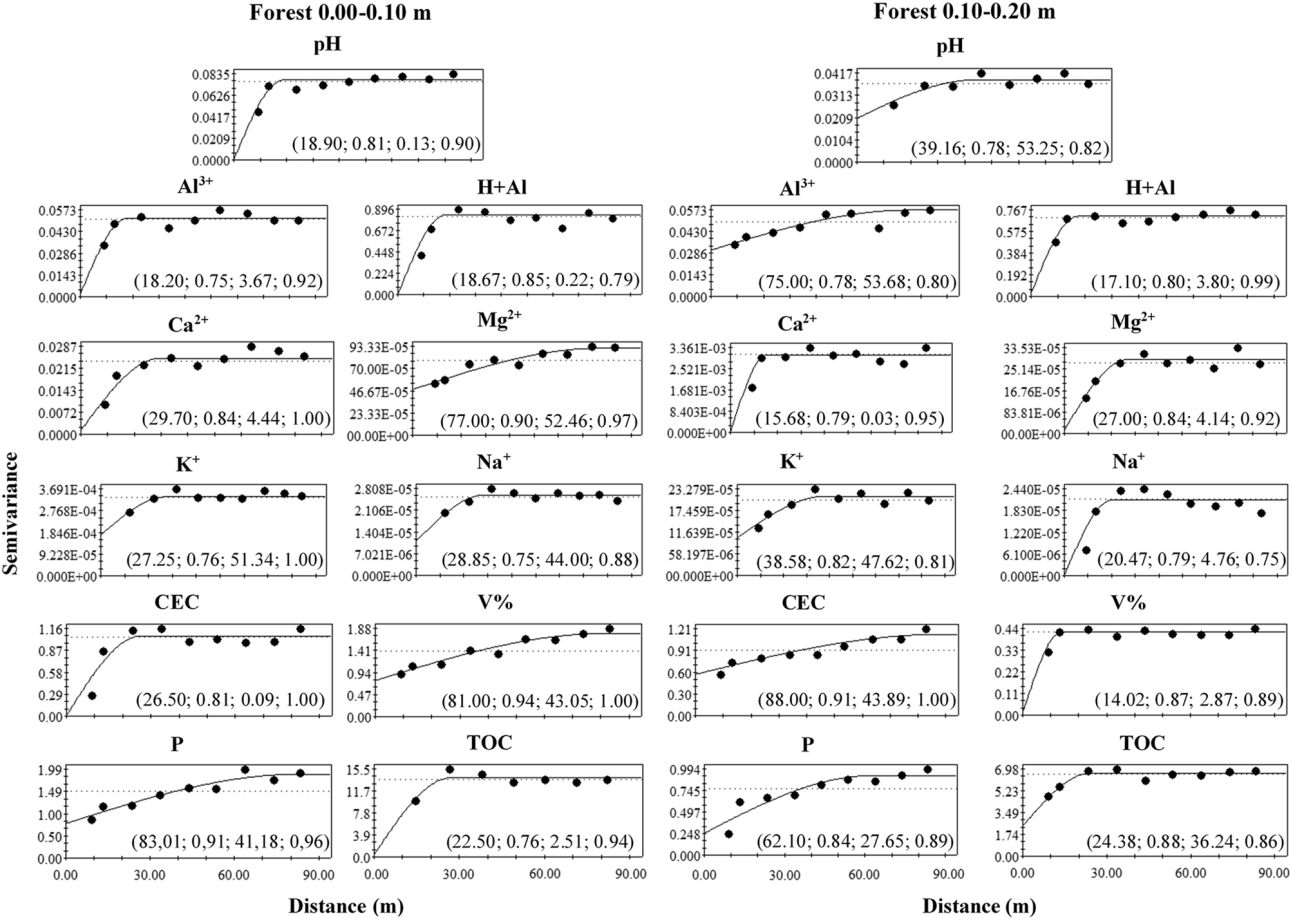
Semivariograms of the chemical attributes of the soil in a forest area in Porto Velho, Rondônia. The values in parentheses are respectively: range, R^2^, Degree of Spatial Dependence (DSD) and cross-validation.

**Figure 7:**
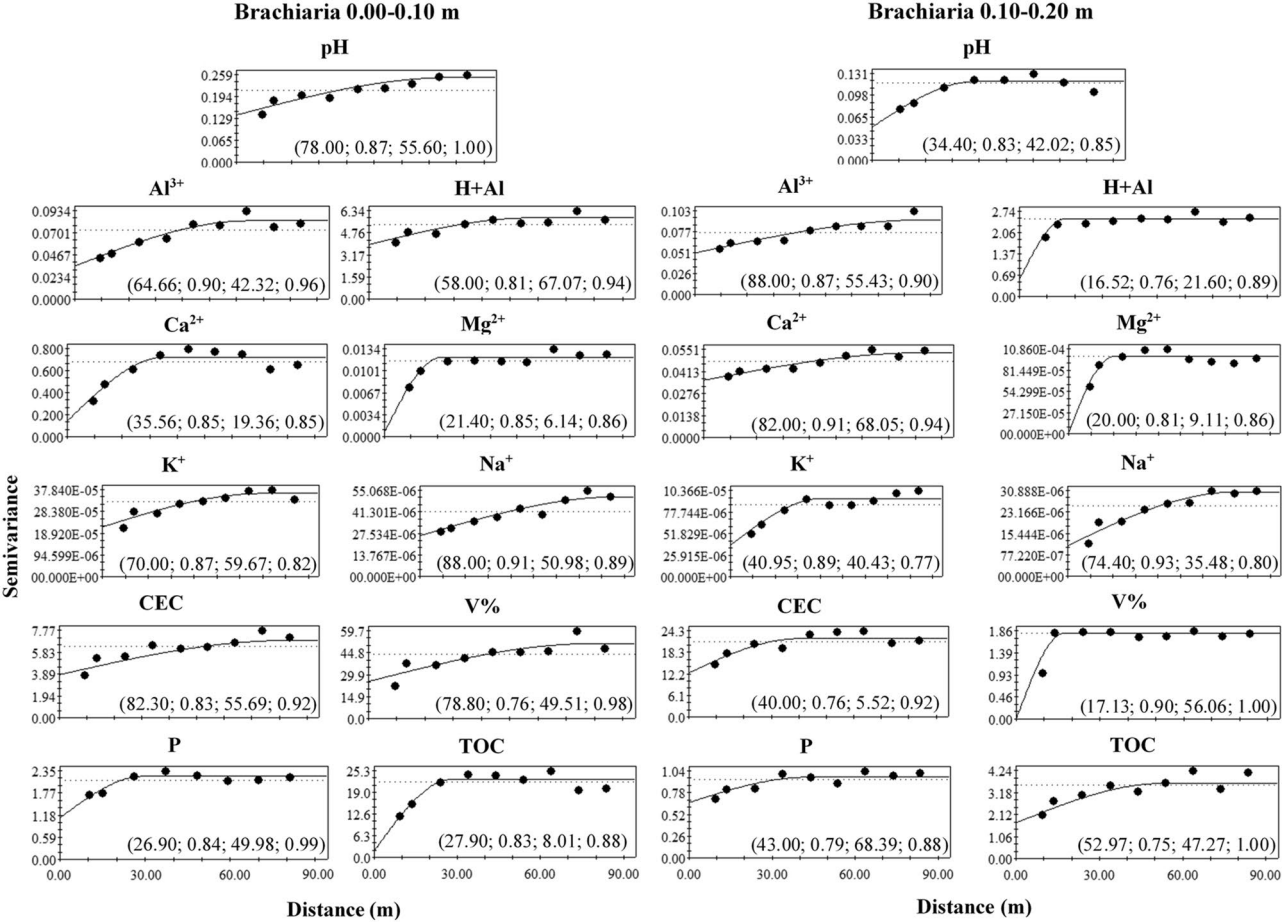
Semivariograms of the chemical attributes of the soil in a brachiaria grass area in Porto Velho, Rondônia. The values in parentheses are respectively: range, R^2^, Degree of Spatial Dependence (DSD) and cross-validation.

**Figure 8:**
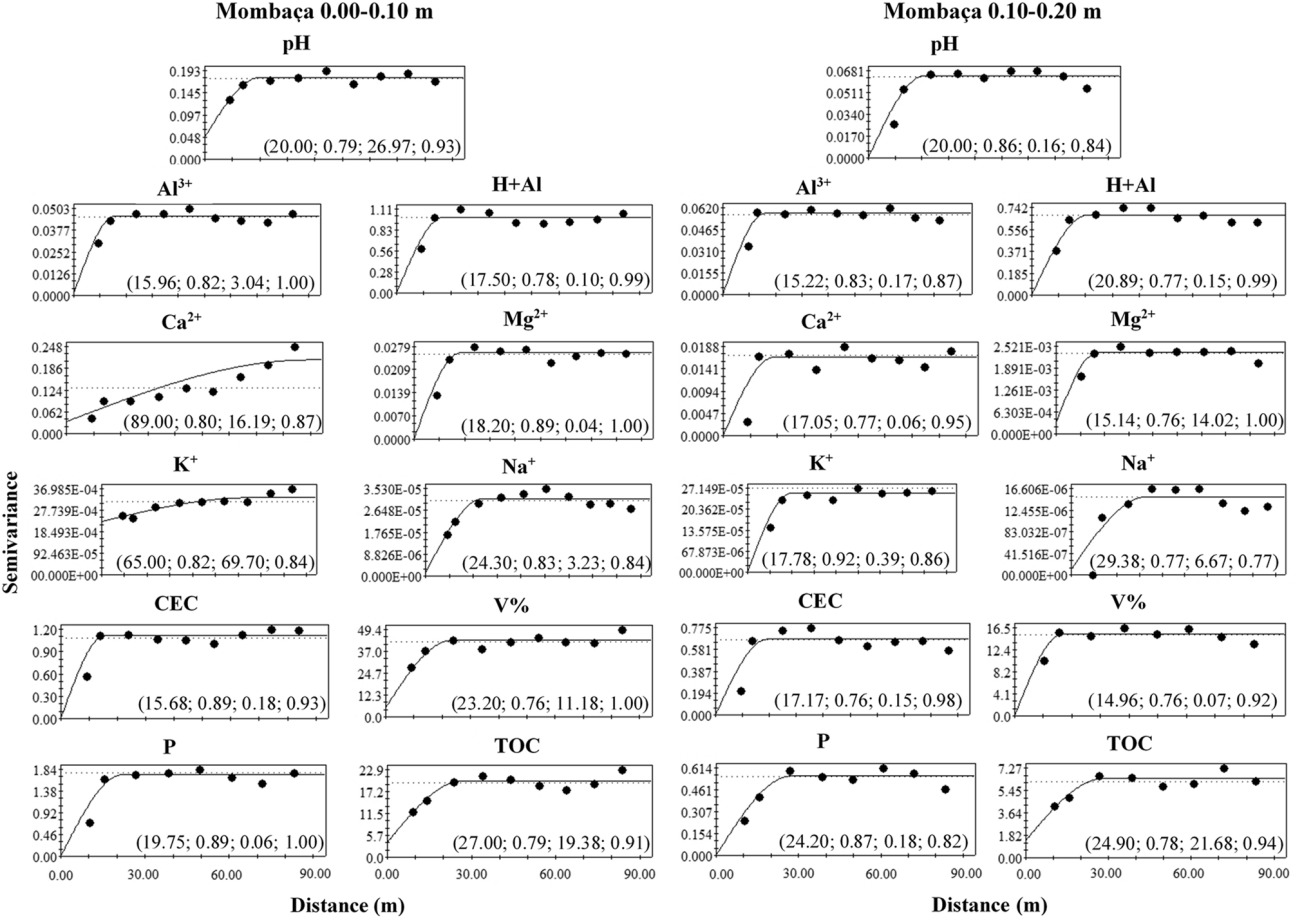
Semivariograms of the chemical attributes of the soil in a mombaça grass area in Porto Velho, Rondônia. The values in parentheses are respectively: range, R^2^, Degree of Spatial Dependence (DSD) and cross-validation.

Figures 6, 7 and 8 show the semivariograms for the forest, brachiaria and mombaça grass areas, respectively. It is observed that all variables showed a spatial dependence structure, with the spherical model being the only one adjusted in all assessed areas and depths. The coefficient of determination (R^2^) showed values ranging from 0.75 to 0.94 while cross-validation (C-V) varied from 0.75 to 1.00 for all areas at the depths studied.

The range indicates the maximum distance at which the attribute is spatially correlated^15^. Range values were observed in the depth of 0.00–0.10 m ranging from 18.20 (Al^3+^) to 83.10 m (P) in the forest area, 21.40 (Mg^2+^) to 88.00 m (Na^+^) for brachiaria and 15.68 (CEC) at 89.00 m (Ca^2+^) for mombaça grass area. At a depth of 0.10–0.20 m, a range ranging from 14.02 (V%) to 88.00 m (CEC) for forest, 16.52 (H + Al) to 88.00 m (Al^3+^) for brachiaria and 14.96 (V%) at 29.39 m (Na^+^) for mombaça grass area.

The Degree of Spatial Dependence (DSD) presented values varying from 0.03 to 69.70% between the studied areas, presenting spatial dependence from strong (DSD < 25%) to moderate (26% > DSD < 75%)^16^, indicating that the variables studied are not randomly distributed in space^17^.

When evaluating the forest area, at a depth of 0.00–0.10 m, it was observed that pH, Al^3+^, H + Al, Ca^2+^, CEC, V and P showed strong DSD while the other attributes showed moderate spatial dependence. At a depth of 0.10–0.20 m, H + Al, Ca^2+^, Mg^2+^, Na^+^ and V% showed strong DSD and the others moderate. In the brachiaria area, at 0.00–0.10 m, only Ca^2+^, Mg^2+^ and TOC showed strong DSD, the other attributes showed moderate DSD. At a depth of 0.10–0.20 m, H + Al, Mg^2+^ and CEC presented strong DSD, the rest of the attributes showed moderate DSD. The area with mombaça grass presented strong DSD for most of the attributes studied at both depths, except for pH and K^+^ at the depth of 0.00–0.10 m, which presented moderate DSD.

**Figure 9:**
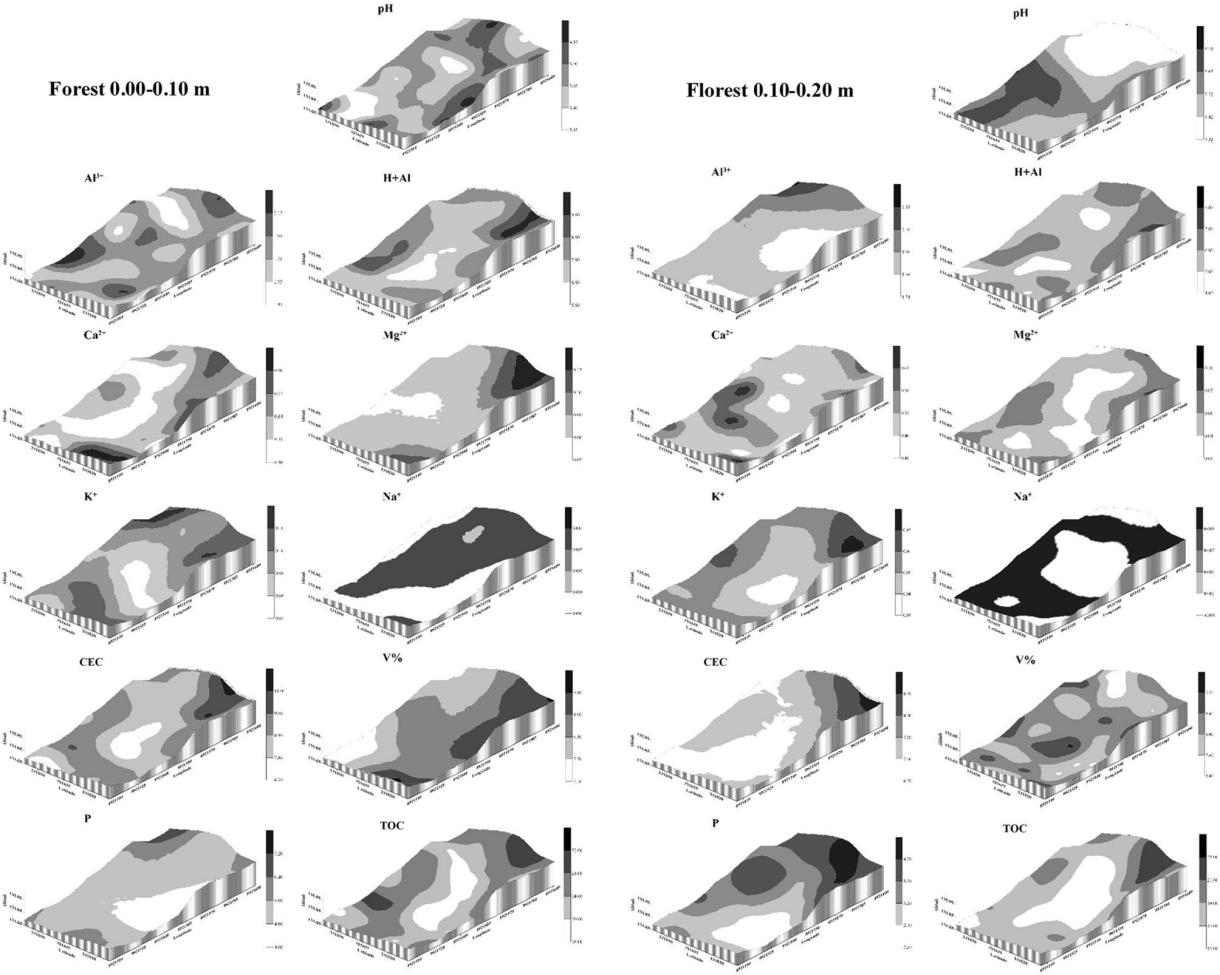
Kriging maps of soil chemical attributes in a forest area in Porto Velho, Rondônia.

**Figure 10:**
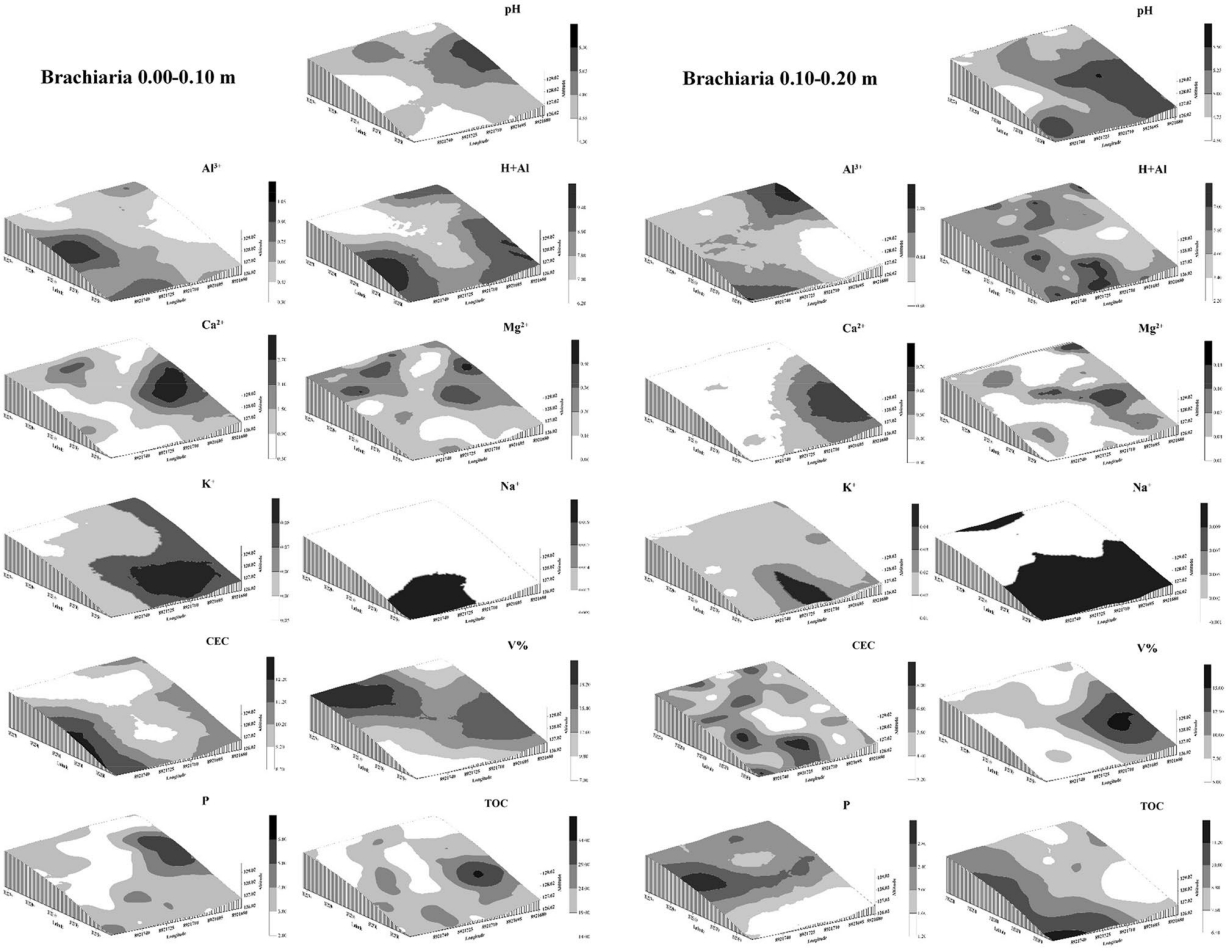
Kriging maps of soil chemical attributes in a brachiaria grass area in Porto Velho, Rondônia.

**Figure 11:**
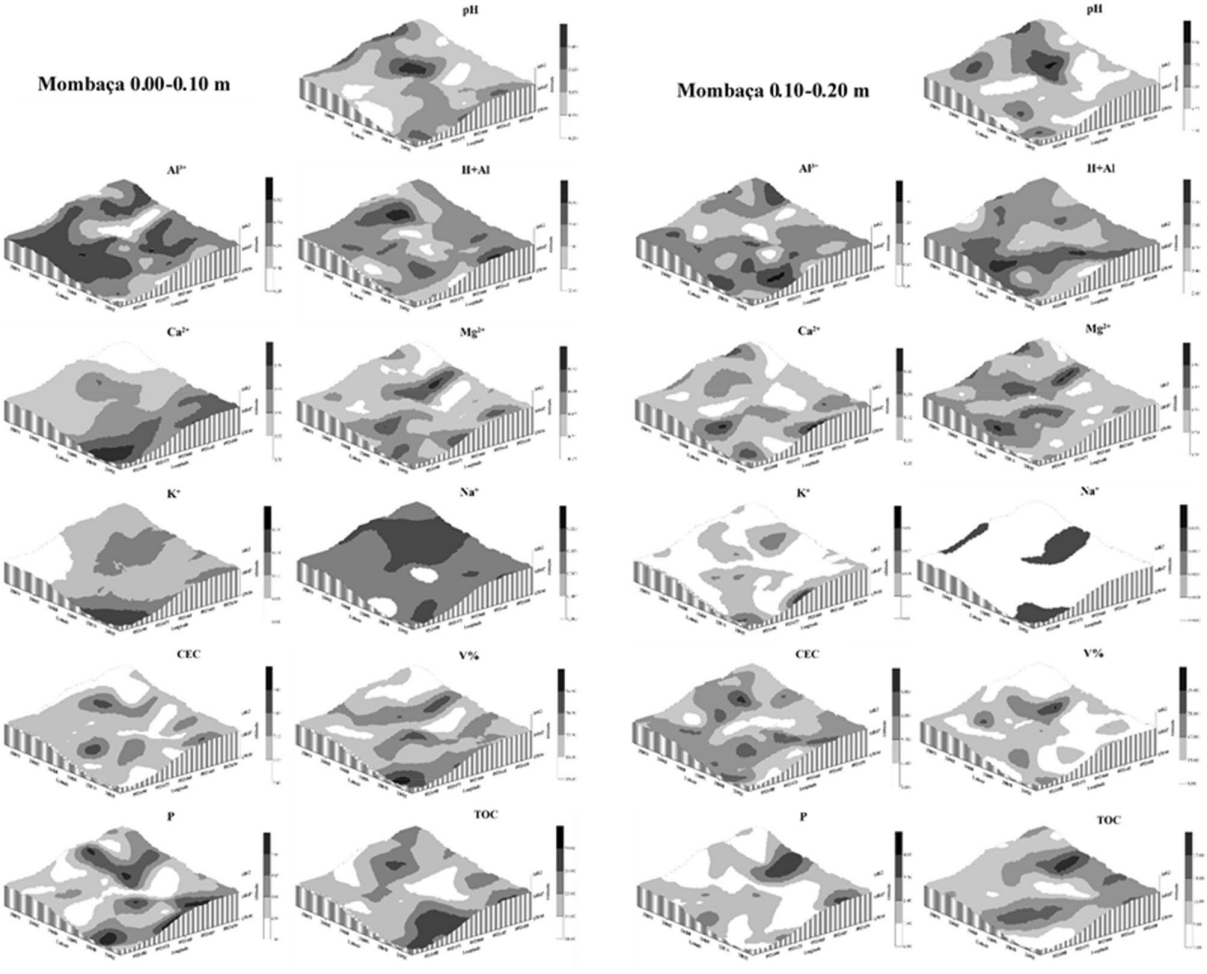
Kriging maps of soil chemical attributes in a mombaça grass area in Porto Velho, Rondônia.

Figures 9, 10 and 11 show the kriging maps for the forest, brachiaria and mombaça grass areas, respectively. It is observed that the mombaça grass area has greater variability in relation to the distribution of attributes compared to the other areas.

**Figure 12:**
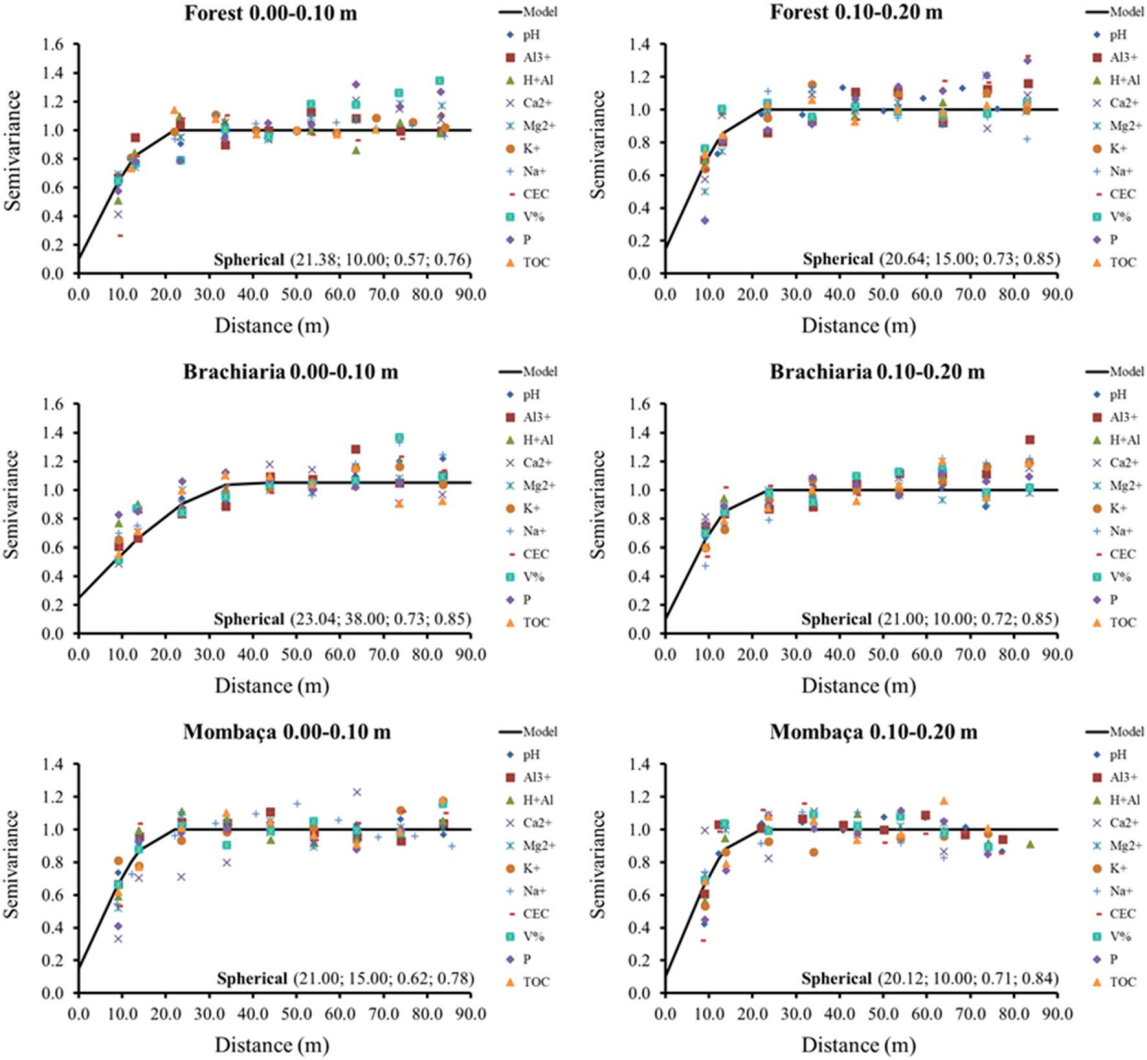
Scaled semivariograms for the environments studied in Porto Velho, Rondônia. The values in parentheses indicate respectively: range, degree of spatial dependence, R^2^ and cross-validation.

The scaled semivariograms for the three studied areas are shown in Fig. 12. The graphs were fitted to the spherical model, which presented R^2^ ranging from 0.57 to 0.73 and cross-validation from 0.76 to 0.85. The studied areas presented range values ranging from 20.12 to 23.04 m, with the highest values found in the brachiaria area and the lowest values in the mombaça grass area. Regarding the DSD, all areas showed strong spatial dependence, except for brachiaria at a depth of 0.00–0.10 m^16^.

Table 4 shows the minimum sample density based on the interval estimated by the staggered semivariograms, for the two depths evaluated in the different environments studied. The area with mombaça grass at a depth of 0.10–0.20 m showed the highest sampling density, with 13 samples ha^−1^, with a spacing of 20 m, however, the forest environment at a depth of 0.00–0 0.10 m showed the lowest value, with 9 samples ha^−1^, with a spacing of 21 m.

**Table 4:** Minimum sampling density and spacing for soil chemical properties in different environments studied in Porto Velho, Rondônia.

Sampling project		Forest	Brachiaria	Mombaça
Sampling density 0.00–0.10 m	points ha^−1^	9	10	12
Sampling density 0.10–0.20 m	points ha^−1^	10	12	13
Spacing 0.00–0.10 m	m	21	23	21
Spacing 0.10–0.20 m	m	20	21	20

**Figure 13:**
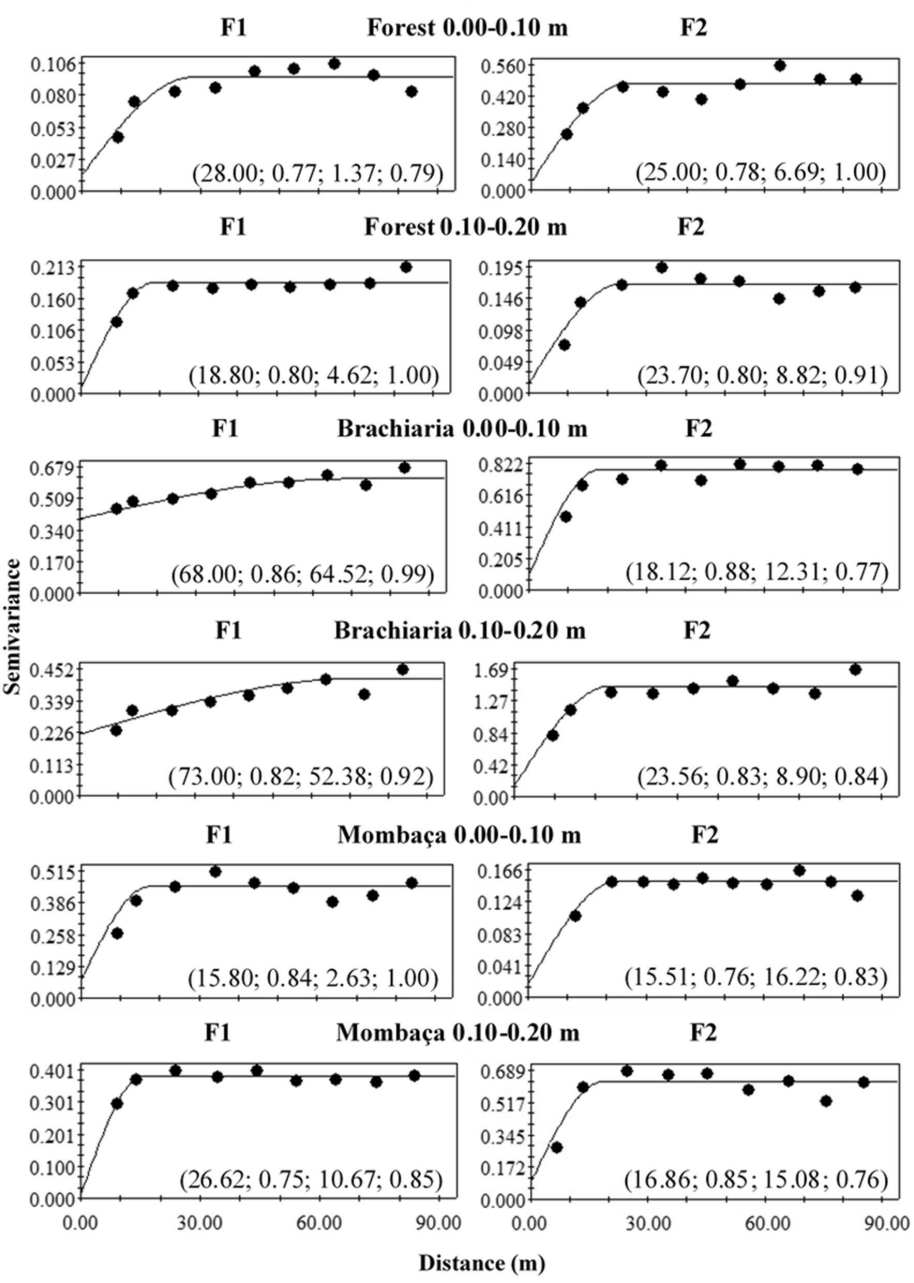
Experimental semivariograms fitted to the principal component scores for the different environments studied in Porto Velho, Rondônia. The values in parentheses are, respectively: range, R2, degree of spatial dependence (DSD) and cross-validation.

Figure 13 shows the semivariogram for the factor score adjustments obtained from the principal component analysis. F1 semivariograms (PC1) for the 0.00–0.10 m depth in the different environments are related to exchangeable bases (Ca^2+^ and Mg^2+^) and to the components of soil acidity (pH and Al^3+^), the F2 semivariograms (PC2) are related to P and TOC. At a depth of 0.10–0.20 m, F1 is related to the components of soil acidity (pH, Al^3+^ and H + Al) and TOC, while F2 is related only to Ca^2+^. In the semivariograms, the adjustment of the spherical model prevailed, which showed R^2^ and cross-validation ranging from 0.76 to 0.88 and 0.76–1.00, respectively. Spatially analyzing, the F1 at the depth of 0.00–0.10 m showed moderate DSD (64.52%) for the brachiaria area and the longest range (68.00 m), while the other areas presented strong DSD. F2 presented a strong DSD for all areas studied, with a greater dependence range for the forest area (25.00 m). The F1 at a depth of 0.10–0.20 m showed moderate DSD for brachiaria area (52.38%), which also had the longest range (73.00 m), the other areas showed strong DSD. F2, on the other hand, presented only strong DSD for all evaluated areas.

## Discussion

When evaluating the components of soil acidity, it is clear that the forested area had higher soil acidity, with high levels of Al^3+^ and H + Al in relation to pastures areas. In their work, Braz et al.^12^ found lower pH values and higher levels of Al^3+^ and H + Al in the forest area in relation to pastures areas and attributed this result to the use of fire to clean the area, which due to the ash, increases the pH and consequently decreases Al^3+^. Silva Neto et al.^13^ explain this mechanism through the occurring fires, of which the ashes along with the bases are incorporated into the soil, dissociating the H^+^ ions from the exchange complex, and the basic cations are adsorbed on the clay surfaces, increasing the pH values. In addition, the vast majority of Amazonian environments are acidic, due to the source material being poor in bases and the high rainfall in the region, which leaches surface nutrients^13,18^. Hong et al.^19^ attribute that the pH variation is according to the predominant plant species. On the other hand, Jeddi and Chaieb^20^ relate the reduction of pH in natural environments due to the greater activity of microorganisms close to the rhizosphere of plants and the release of root organic acids. In relation to Al^3+^, the high levels found in the forest area may be due to the low input of production of organic compounds capable of complexing aluminum^21,22^.

The high levels of exchangeable bases in the pastures areas in relation to the forest corroborate with Braz et al.^12^, who found higher levels of Ca^2+^ and K^+^ in pasture area. In their work, Silva Neto et al.^13^ observed higher levels of cations in burnt areas compared to the forest, the same authors attributed the high levels of bases to the ashes deposited after the burning.

In the work of Araújo et al.^9^, analyzing the forest-pasture conversion, they found low levels of P in the first depths of the soil. Lisbôa et al.^10^, studying soil quality indicators related to pasture degradation in Latossolo, found that soil organic carbon is one of the variables sensitive to changes in management. The accumulation or release of TOC to the atmosphere depends on the type of management applied to the soil and forage, where pastures cultivated in fertile soils cause the accumulation of C. In contrast, when cultivated in soils with low fertility, the implementation of pastures results in losses of C in the soil. However, the magnitude of losses depends on the degree of pasture degradation^23^. The stabilization of organic carbon in soils under different types of management depends on the amount of carbon stored in the soil and the type of structure that is deposited^24^.

The lower levels of P and higher TOC in the mombaça grass area in relation to brachiaria are due to the higher nutritional requirement of mombaça grass in relation to the brachiaria, which in addition to requiring more nutrients, produces more biomass that contributes to greater TOC deposit in the soil^25,26^.

The interactions between chemical attributes reflect the ability of one variable to interfere with the other directly or antagonistically. The interactions between pH with other variables were also observed by Braz et al.^12^, who studied the effect of converting forest to pasture and observed that, with an increase in pH, there is greater availability of nutrients (exchangeable bases) in the soil and a decrease in the content of exchangeable Al^3+^ in pasture areas. According to Perin et al.^27^, the increase in pH causes a decrease in the Al^3+^ content due to its precipitation, which consequently decreases the H + Al content.

TOC is one of the attributes related to organic matter in the soil that most influences the chemical quality of the soil. In their work, Petter et al.^28^ showed that there is a positive correlation between TOC and CEC. This is due to the ability of organic matter to supply loads and nutrients to the soil, as the humic fraction of organic matter can present 200–500 cmol_c_ kg^−1^ of negative load on the soil^29^. This promotes the adsorption of exchangeable bases, keeping them in the soil exchange complex and preventing them from being lost by leaching^30^. In their study, Galang et al.^31^ showed that the stocks of inorganic P in the superficial layers of the soil is due to the conversion of organic P, and this conversion decreases with increasing temperature and time. Often the TOC is related to soil acidity, especially in the Amazon region where there is high biological activity, which combined with a hot and humid environment, promotes greater production of CO_2_ in the soil solution, which acts as a source of soil acidity^13^. However, organic matter has the ability to complex aluminum due to the production of organic compounds resulting from its decomposition^21,22^, a process that can be intensified in pasture soils due to greater exposure to climatic factors.

In both depths, the pastures environments differed from the forest environment. We attribute this to the use of fire to clean the areas, which due to the increase in nutrients present in the ash into the soil provided a higher pH and the availability of nutrients^2^. However, when converting to pastures, due to Amazonian soils being naturally acidic, it is necessary to adopt corrective practices and improve the supply of nutrients^32^.

The CV allows comparing the variability between samples of variables with different units, but it does not allow analyzing the spatial variability of soil attributes or their spatial pattern^33^. Pasture areas (brachiaria and mombaça grass) presented higher CV than the forest area. This variation in the higher CV values in the pastures areas can be attributed to the complex interactions of the formation processes and the management practices adopted in the soil and in the crops^34^.

In their work, Oliveira et al.^33^ studied soils in Amazonas and observed the predominance of the spherical model in the adjustment of semivariograms for soil attributes of forested areas and the exponential model in pasture. However, in this work, the predominance of the spherical model is observed, indicating that the attributes have high spatial continuity and are less erratic over short distances^35^.

The area with brachiaria showed the highest range values for most of the variables studied in relation to the other areas, indicating that the area of brachiaria has less variability and is more homogeneous. On the other hand, the area with mombaça showed the lowest range values, indicating that the chemical attributes of this area have greater variability and their distribution is more heterogeneous. This greater variability in the mombaça area may be related to the greater grazing intensity, which corresponds to 6.14 animails ha^−1^ in relation to the brachiaria area, which is 4.50 animals ha^−1^. The higher grazing intensity related to grazing preference zones^36^, with local microrelief that directs the flow of water and nutrients present in the soil^37^, contribute to the generation of variability of chemical attributes in pasture areas.

Oliveira et al.^33^, studied different soil management in southern Amazonas, the authors found strong DSD in the forest area and moderate in the pasture area with brachiaria for chemical attributes at a depth of 0.00–0.20 m. The area of mombaça grass showed a strong DSD for most variables in relation to the forest and brachiaria environments, showing that the chemical attributes in this cultivation environment are more influenced by the intrinsic properties of the soil, linked to formation factors such as source material, relief and climate^16^.

As a product of geostatistics, we have kriging maps that allow the observation of the distribution of the studied attributes. With this, it is possible to establish criteria for use and management in an isolated way, allowing rationing of management in relation to fertilization and chemical correction of the soil, distributing nutrients in areas that need it most^36^. This makes it possible to reduce production costs, increase productivity and reduce the risks of environmental pollution with the excessive use of chemical fertilizers^38^.

Management zones are sub-areas of a larger parcel where soil attributes can be considered homogeneous, they take into account not only the same soil class, but also the spatial variability existing within the same soil class^39^. Therefore, the management zone can be defined within the same soil class from grid soil samples^40^, used to develop semivariograms. Multivariate analyzes, mainly those of principal components, have been used to synthesize new variables based on the interaction of several original variables, and thereby differentiate zones within the same soil management systems^41^.

**Figure 14:**
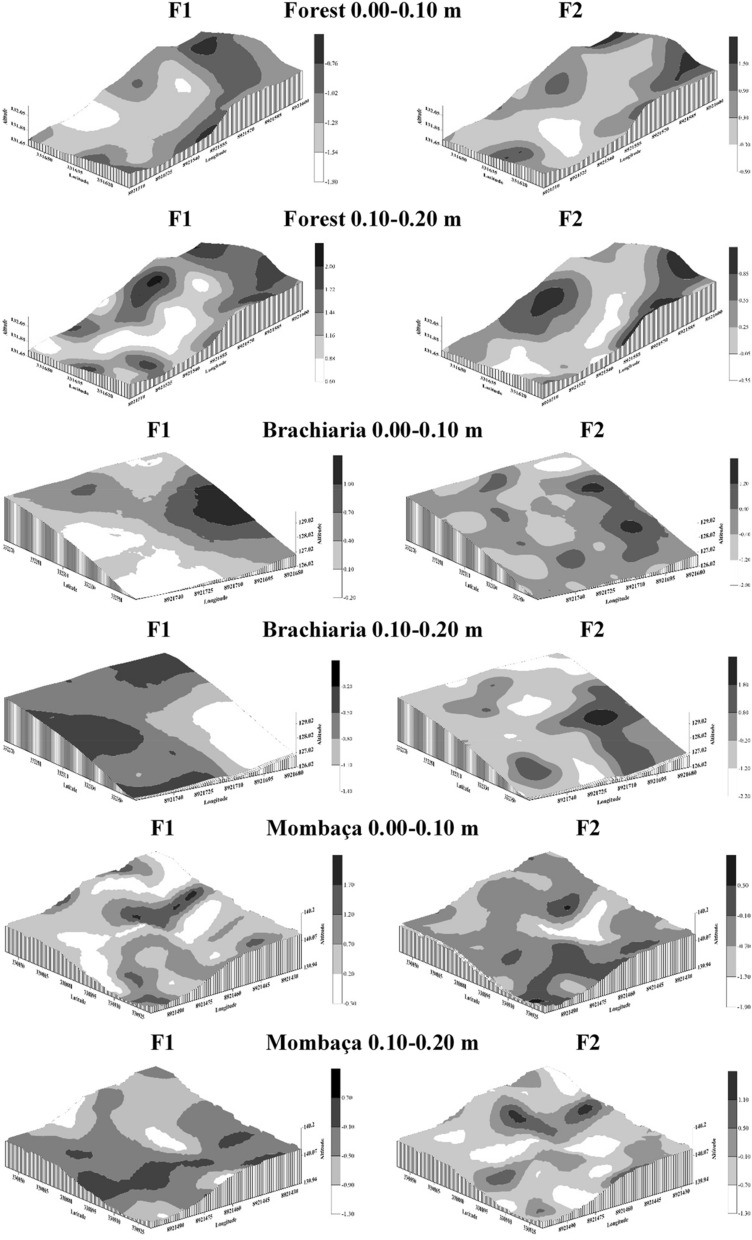
Kriging maps of the principal component scores for the different environments studied in Porto Velho, Rondônia.

When evaluating the kriging maps of the scores (Fig. 14), the formation of five management zones is observed. For Burak et al.^42^, the higher the scores, the greater the contributions to positive correlations between variables that make up each factor. In contrast, the lower the scores, the greater the contributions to negative correlations. Thus, when looking at the F1 maps at a depth of 0.00–0.10 m, it is evident that most of the scores are positive for pastures areas, while in the forest area they presented higher negative scores. The areas with positive scores indicate a direct correlation between pH and bases (Ca^2+^ and Mg^2+^), but the areas with the highest negative scores are due to Al^3+^, which is less available as the pH and bases increase. For F2, the forest area showed most of the positive scores, while the pasture areas showed the most negative. This shows that the availability of P is correlated with the increase in TOC in the forest area, and in pasture environments, this happens less frequently. For a depth of 0.10–0.20 m, F1 in the forest area showed most of the positive scores, while the pasture scores showed most of the negative scores, indicating that the TOC in the forest is correlated with the acidity components, however, this correlation occurs less frequently in pastures. These observed correlations show that each zone in each environment needs specific, more or less intensive management, and with this increase the efficiency of the use of natural resources, reduce the impact of agriculture on the environment and optimize the economic costs for the cultivation system^38,43^.

The conversion of forest into pasture by fire provided an increase in pH and exchangeable base values, reducing exchangeable aluminum contents and potential acidity, however, it induces losses of phosphorus and organic carbon in the soil.

Through the multivariate analysis, the formation of two groups was observed, the forest and the pastures. The forest environment is characterized by being more acidic, having higher levels of P, total organic carbon, exchangeable aluminum and potential acidity, while pasture environments are characterized by having a higher pH and higher levels of exchangeable bases. Among pasture environments, mombaça grass showed higher fertility.

The mombaça grass showed greater spatial variability of chemical attributes, which was attributed to greater grazing intensity and to the microreliefs that direct the flow of water and nutrients.

The variability in the areas generated specific management zones for the components of acidity, exchangeable bases, phosphorus and organic carbon of the soils. In these environments, it is necessary to establish specific management zones, more or less intensive, for greater efficiency in the use of natural resources, reduction of the impact of agriculture on the environment and optimization of economic costs.

## Material and methods

### Characterization of the study area

**Figure 15:**
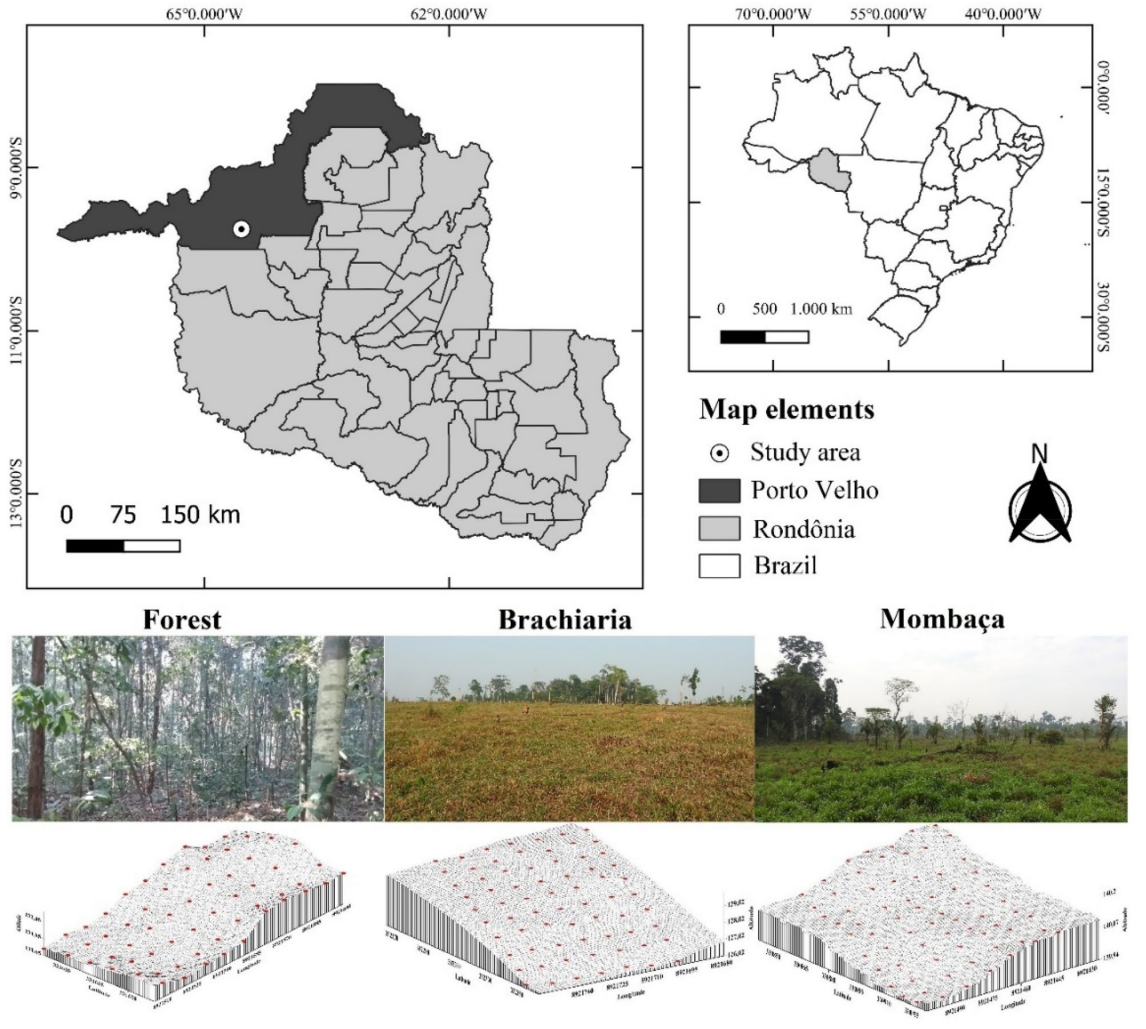
Location and elevation map of the study areas.

The study was carried out in the União Bandeirantes district located in the municipality of Porto Velho, Rondônia, Brazil (Fig. 15), in the year 2019, from September to October, at the end of the dry season, in three areas, two areas cultivated with brachiaria grass (*Brachiaria brizantha* cv. Marandu) and mombaça grass (*Panicum maximum* cv. Mombaça) and an area of native forest. The study areas are located at coordinates 9°45′41.21″ south and 64°31′43.18″ west for brachiaria, 9°45′9.67″ south and 64°32′5.88″ west for forest and 9°45′ 12.29″ south and 64°32′30.11″ west to mombaça.

The region's climate belongs to group A (Tropical Rainy Climate) and Am type (monsoon rains), with a short dry season between June and September. The average annual rainfall ranges from 2500 to 2800 mm. The annual average temperature is between 24 and 26 °C. The relative humidity is quite high, varying between 85 and 90% in the rainy season and between 60 and 70% in the dry season^44^. The vegetation typology is called dense ombrophilous forest, consisting of dense and multi-stratified trees from 25 to 30 m high^45^.

The study area is on the trays of the Amazonian depression, the regional relief has a smooth wavy surface, with an altitude ranging from 100 to 200 m. The soils found are developed from undifferentiated sedimentary covers, associated with environments of alluvial fans, fluvial channels, flood plains and lakes, constituted by sediments whose granulometry varies from gravel to clay, with significant lateritization^46^.

All soils have local relief varying from flat to smooth wavy and well drained. They are classified as “Latossolo Amarelo Distrófico típico, A moderado, textura argilosa” according to the Brazilian System of Soil Classification^47^ (Table 5).

**Table 5:** Physical attributes of the soil profile in Porto Velho, Rondônia.

Horizon	Depth	Gravel	ADFE	Total sand	Coarse sand	Thin sand	Silte	Clay	CDW	DF	S/C	SD	PD	TP
	cm	g kg^−1^								%		g cm^−3^		m^3^ m^−3^
**Latossolo Amarelo Distrófico típico, A moderado, textura argilosa**														
A	0–10	11	989	644	470	174	34	322	256	20	0.11	1.16	2.74	0.58
BA	10–25	9	991	568	371	197	34	398	341	14	0.09	1.45	2.83	0.49
Bw1	25–45	12	988	528	345	183	25	447	391	13	0.06	1.37	2.72	0.50
Bw2	45–71	11	989	520	350	170	23	457	0	100	0.05	1.47	2.90	0.49
Bw3	71–95	15	985	530	345	185	28	442	0	100	0.06	1.38	2.83	0.51
Bw4	95–122	18	982	529	361	168	48	423	0	100	0.11	1.39	2.90	0.52
Bw5	122–150 +	13	987	483	302	181	46	471	0	100	0.10	1.43	2.90	0.51

*ADFE* Air-dried fine earth, *CDW* Clay dispersed in water, *GF* Degree of flocculation, *S/C* Silt/clay, *SD* Soil density, *PD* Particle density, *TP* Total porosity.

The areas were cleaned using a chainsaw and fire, and then the sowing of forages was carried out. It is important to clarify that liming and fertilization were never carried out. Altogether there are 110 animals bred across the 44.28 ha area, which alternate between the paddocks of brachiaria and mombaça grass every 45 days. The area with brachiaria was implanted in 2008 with an extension of 26.36 ha, remained unused for one year and was used infrequently until 2010, after which 4.5 animals per ha were used. The area with mombaça grass has 17.92 ha, was introduced in 2007, was left unused for three years, after which 6.14 animals per ha were used in the 45-day rotation between the brachiaria.

### Field methodology

In the paddocks where the pastures were being restored after grazing, study meshes of 90 × 60 m grid was established for both areas with brachiaria and mombaça grass, and 90 × 50 m for the forest area with a regular spacing of 10 m between the sample points. The soils were sampled at the crossing points of the meshes, under the depths of 0.00–0.10 and 0.10–0.20 m, making a total of 60 sample points for forest area and 70 points for pasture areas, totaling 400 samples.

The crossing points of the meshes were georeferenced with a GPS device (DATUM SIRGAS 2000) for the construction of the Digital Elevation Model (MDE). At the same points sampled with the aid of precision level and sight (ruler), the altimetric survey was carried out for the purpose of studying the relief in the meshes.

To obtain the samples at the crossing points of the meshes, samples were collected in each area with a preserved structure in the form of 10 cm high clods. These samples were used to determine the chemical properties of the soil.

### Laboratory analysis

After the soil had gone through the shade drying process and sieved in a 2 mm mesh, characterizing an air-dried fine earth (ADFE), chemical analyzes were carried out according to the methodology proposed by Teixeira et al.^48^. The following were determined: pH in water, potential acidity (H + Al), exchangeable aluminum (Al^3+^), calcium (Ca^2+^), magnesium (Mg^2+^), phosphorus (P), potassium (K^+^) and total organic carbon (TOC).

The pH in water was determined potentiometrically, using a pHmeter in 1:2.5 soil/water ratio.

Calcium (Ca^2+^), magnesium (Mg^2+^) and exchangeable aluminum (Al^3+^) were extracted by KCl 1 mol L^−1^ solution. The contents of Al^3+^ were determined by titration, using NaOH 0.025 mol L^−1^ and bromothymol blue as a colorimetric indicator. The levels of Ca^2+^ and Mg^2+^ were determined by atomic absorption spectrometry.

Potential acidity (H + Al) was extracted with calcium acetate buffered at pH 7.00 and determined titration using NaOH 0.025 mol L^−1^ and phenolphthalein as an indicator.

Potassium and available phosphorus were extracted by *Mehlich-1*. The levels of P were determined by UV–Vis spectrophotometer and the levels of K^+^ by flame spectrophotometry.

Based on the determinations of exchangeable cations and potential acidity, potential cation exchange capacity (CEC) and base saturation (V%) were calculated.

Total organic carbon (TOC) was determined using the Walkley-Black^49^, method, modified by Yeomans and Bremner^50^.

In order to physically characterize the studied soil, granulometric, texture, density and porosity analyzes were performed^48^. The granulometry was determined by sieving the soil after drying and separating the gravel and air-dried fine earth (ADFE) fractions. The soil texture was determined by the pipette method, with NaOH 1 mol L^−1^ solution as a chemical dispersant and mechanical agitation using the Wagner type agitator, in a slow rotation apparatus for 16 h at 50 rpm. The coarse and fine sand fraction were separated by sifting, and the clay and silt fractions were separated by sedimentation. Additionally, the analysis of clay dispersed in water (CDW) was performed, and then the degree of flocculation (GF) was calculated through the proportion of the difference between the total clay and the clay dispersed in water (CDW). The soil density (SD) was obtained by the method of the volumetric ring and the particle density (PD) by the method of the volumetric balloon. Total porosity (TP) was calculated from data on soil and particle densities, using the following equation: TP = (1 − SD/PD).

### Uni, bi and multivariate statistical analysis

After determining the chemical attributes, the data were submitted to univariate, bivariate and multivariate analyzes using the statistical software Statistica 7^51^.

Univariate statistic was assessed using descriptive statistics, calculating the mean, median, coefficient of variation, skewness and kurtosis. In addition, boxplot graphs were made in order to represent descriptive statistics and primary data distribution. The hypothesis of data normality within each environment was examined using the Kolmogorov–Smirnov (KS) test. Then, ANOVA analysis of variance was performed and, when it was significant, comparisons of the means of the variables between the three studied environments were performed, using the Tukey test at 5% probability.

By the Kolmogorov–Smirnov test at 5% probability, normality was observed in the data distribution for most of the variables studied, except for Na^+^ in the areas of forest and mombaça grass (and brachiaria in 0.10–0.20 m), for Al^3+^ at the depth of 0.00–0.10 m in the areas of brachiaria and mombaça grass, for Ca^2+^ and Mg^2+^ at the depth of 0.10–0.20 m in the area of brachiaria, and for K^+^ at the depth of 0.10–0.20 m from the brachiaria and mombaça areas (Table 1).

This normality is also observed for the mean and median close to each other (Figs. 1, 2, 3 and 4). The skewness of the data showed values around − 0.51 to 1.33 (Table 1). Skewness values greater than zero were found for V% at the depth of 0.00–0.10 m and Na^+^ at the depth of 0.10–0.20 m in the forest area, pH and TOC at the depth of 0.10–0.20 m in the brachiaria area and Al^3+^ in the mombaça area, indicating that the distribution is asymmetric on the right. The other attributes evaluated for depths and areas showed an asymmetric distribution on the left^52^. For kurtosis values ranging from − 1.49 to 1.63 were observed, these should preferably be null, however values between − 2 and + 2 are acceptable^53^.

Bivariate statistics was used to verify correlations between the variables studied, pair by pair, in order to study the direct or antagonistic influence of one on the other. Pearson's correlation test at 5% probability was used in the evaluation.

In the multivariate analysis, a factor analysis of the principal components (PC) was carried out in order to find statistical significance of the sets of chemical attributes of the soil that most discriminate the environments, with respect to the different areas under study, obtained as an answer which environments suffer the most influence in its chemical attributes by anthropic action.

The adequacy of the factor analysis was made by the Kaiser–Meyer–Olkin (KMO) measure, which assesses the simple and partial correlations of the variables, and by the Bartlett sphericity test, which is intended to reject the equality between the correlation matrix with identity. The extraction of the factors was performed by the analysis of the main component, incorporating the variables that presented commonality equal to or greater than five (5.0). The choice of the number of factors to be used was made by the Kaiser criterion (factors with eigenvalues greater than 1.0). The orthogonal rotation (varimax) was performed in order to simplify the factor analysis, and representing it in a factorial plane of the variables and scores for the main components^54^.

### Geostatistical analysis

Geostatistics was used to assess the spatial variability of the chemical attributes of the soil and the scores obtained by factor analysis. To perform the geostatistical analysis, it was necessary to know whether there is spatial dependence or not on the attributes studied, verified through the graph of the semivariogram. Based on the stationary assumption of the intrinsic hypothesis, which the semivariogram was estimated by:

1 $${{\hat{\text{y}}(h) = 1}}/{2}\eta (h)\sum\nolimits_{i = 1}^{\eta (h)} {\left[ {{\text{Z (}}x_{i} {) } - {\text{ Z(}}x_{i} + h{)}} \right]^{{2}} }$$

being *ŷ*(*h*) = value of the semivariance for a distance *h*; $\eta$(*h*) = number of pairs involved in calculating the semivariance; *Z*(*x*_*i*_) = value of attribute *Z* in position *x*_*i*_; *Z*(*x*_*i*_ + *h*) = value of attribute *Z* separated by a distance *h* from position *x*_*i*_.

The adjustment of semivariograms were made based on the best coefficient of determination (R^2^) and cross-validation (C-V), estimated by the Software GS + 7.0^55^. From these adjustments, the coefficients of the theoretical model for the semivariogram were defined: nugget effect (C_0_) = value of the semivariance for the zero distance, which represents the random variation component; structural variance (C_1_); sill (C_0_ + C_1_) = value of the semivariance in which the curve stabilizes over a constant value; and range (a) = distance from the origin to where the sill reaches stable values, expressing the distance beyond which the samples are not correlated^56^.

For the analysis of the degree of spatial dependence (DSD) of the attributes under study, the classification by Cambardella et al.^16^ was used, in which the soil properties are considered with strong spatial dependence if the ratio of the nugget effect (C_0_) to the sill (C_0_ + C_1_) is less than 25%. If the ratio is between 26 and 75%, the spatial dependence is considered moderate, whereas if the soil property is greater than 75% to approximately 95%, they are classified as weak spatial dependence.

After adjusting the permissible mathematical models, the data were interpolated using kriging in the Surfer software version 13.00^57^. The construction of Krigagem maps made it possible to verify and spatially relate chemical attributes.

In this work, scaled semivariograms for all variables in each area and depth studied were used in order to reduce them to the same scale, facilitating the comparison between results from different areas^58^. The experimental semivariograms were scaled by dividing the semivariances by the statistical variance^59^. The choice of the scaled semivariogram model that best fitted the data was made based on R^2^ and C-V, in addition to practical knowledge of the behavior of attributes in the environments.

Through the reach of the scaled semivariograms, the minimum number of samples was determined so that it is possible to promote the characterization of the spatial variability of the attributes studied in each area and depth^60^.

2 $${\text{N}} = A/{\text{(a}}^{{2}} /{10000)}$$

being *N* = minimum number of samples required to determine a sampling mesh; *A* = total area, in ha; *a* = range of the semivariogram, in m.

## Data Availability

The PDIR-CDR dataset generated and analyzed during the current study is available from the corresponding author on reasonable request.
